# Availability and advertising of sugar sweetened beverages in South African public primary schools following a voluntary pledge by a major beverage company: a mixed methods study

**DOI:** 10.1080/16549716.2021.1898130

**Published:** 2021-04-29

**Authors:** Agnes Erzse, Nicola Christofides, Nicholas Stacey, Kelsey Lebard, Louise Foley, Karen Hofman

**Affiliations:** a SAMRC/Centre for Health Economics and Decision Science, PRICELESS, Faculty of Health Sciences, University of Witwatersrand School of Public Health, Johannesburg, South Africa; b Faculty of Health Sciences, University of Witwatersrand School of Public Health, Johannesburg, South Africa; c MRC Epidemiology Unit, University of Cambridge, UK

**Keywords:** Sugar-sweetened beverage, voluntary pledge, food environment, primary school, South Africa

## Abstract

**Background**: Towards the end of the 2017 school year, a prominent beverage company in South Africa pledged to remove their sugar-sweetened beverages (SSBs) and advertisements from primary schools in order to contribute to the realization of a healthy school environment.

**Objectives**: To assess the availability and advertising of the company’s beverages in public primary schools in Gauteng province following their voluntary pledge to remove the products, and to explore perceptions of school staff regarding SSB availability in schools and processes related to the implementation of the pledge.

**Methods**: In 2019, we conducted a representative survey of public sector primary (elementary) schools in Gauteng province, South Africa. A random sample of schools was drawn, with schools stratified by whether or not they charge fees. This was a proxy for the socioeconomic status of the locale and student body. At each school, the availability of beverages and presence of advertising or not was assessed by an observational audit tool and differences across fee status assessed by Pearson $\chi^{2}$ test. Semi-structured interviews were conducted with a purposive sample of school officials. Data from the interviews were coded and thematic analysis conducted.

**Results**: Two years following a voluntary pledge, the company’s carbonated SSBs were available for sale in 54% (CI: 45–63%) of schools with tuck shops and advertised in 31% (CI: 25–39%). Qualitative interviews revealed a complex landscape of actors within schools, which, combined with indifference or resistance to the pledge, may have contributed to the continued availability of SSBs.

**Conclusions**: Though we were unable to examine SSB availability before and after the pledge, our findings provide some preliminary evidence that voluntary pledges by commercial entities are not sufficient to remove SSBs and advertisements from schools. Mandatory regulations coupled with in-depth engagement with schools may be an avenue to pursue in the future.

## Background

The consumption of sugar-sweetened beverages (SSBs) is a significant source of additional caloric intake and contributor to the onset of obesity. Evidence from systematic review indicates that increased SSB intake promotes weight gain and the development of obesity especially in children [1]. Over the past decade, South Africa shows increasing prevalence of obesity among adults and children. Data from 2016 indicate that 13% of children under 5 were overweight or obese, which is more than twice the global average of 6% [2,3]. Childhood obesity increases the risk of obesity-related non-communicable diseases (NCD) such as diabetes, heart disease and cancer in later life [4–6]. Children spend significant time in schools during developmentally important phases, and in many Sub-Saharan African countries, children consume up to 20–30% of their total daily caloric intake from school meals [7,8]. Therefore, school is one of the several critical environments in which to address childhood obesity [9].

The World Health Organization (WHO) guidelines for intake of free sugars (including fruit juices) is less than 5% daily calories, or 19 g per day for four to six years old and 24 g for seven to ten years old [10]. South African children consume an average of 50 g sugar per day [11], significantly exceeding these recommendations. A major source of added sugars is ultra-processed foods whose manufacturers are incentivized to pursue sales, which may not align with health concerns, particularly among vulnerable populations such as children [12]. Children can easily obtain SSBs though, informal vendors, canteens, and school tuck shops, which are designated areas within the school premises that have food and beverage items available for sale to students before, during or after school [13–15]. Additionally, school children are frequently exposed to harmful marketing of unhealthy foods and beverages. In the Western Cape province, over 60% of primary schools displayed signage on school premises that advertised well-known beverage companies [16]. Systematic reviews of evidence consistently demonstrated that unhealthy food marketing is important contributor to childhood obesity, with attendant effects across the lifespan [17,18].

The South African Government recognizes the evidence of the harmful effect of marketing and consumption of unhealthy products by children. It promotes the implementation of the WHO Set of recommendations on the marketing of foods and non-alcoholic beverages to children, including SSBs [19]. Furthermore, it has committed to the prevention and control of obesity through its national strategic plans [9,20] and the creation of a healthy school food environment through the National School Nutrition Programme (NSNP) [21]. A significant public health milestone in the response to the country’s obesity epidemic was the introduction of a tax on SSBs in 2018 [22]. Following the announcement of the SSB tax policy in 2016, a series of voluntary measures were proposed by beverage industry actors including the reduction of container sizes, as well as limiting the supply and advertisement of SSBs in primary schools [23–25].

In August 2017 Coca-Cola Beverages South Africa (CCBSA) made an announcement that it would restrict the supply and advertising of their SSB brands to primary school outlets in the country. The announcement took the form of a letter addressed to primary schools that were CCBSA customers. Three main actions were outlined in the pledge. Firstly, CCBSA would no longer supply primary schools with their products that contain added sugar. Secondly, it would extend the availability of low and no-sugar options, as well as promote portion control with smaller pack sizes. Thirdly, it would remove all branding and advertising within the school premises and replace those with generic red boards with white writing. The pledge thus allowed the continued sale of zero-sugar products (hereafter referred to as artificially sweetened beverages, ASB), non-flavoured water, and 100% fruit juice. The pledge did not allow the sale of flavoured water, which has added sugar, nor other carbonated- and non-carbonated sugary drinks. The pledge was aimed at ‘playing an active role in addressing rising obesity rates in South Africa, especially among children’, and contributing to ‘government objectives’ [26].

In the context of this voluntary pledge by CCBSA, we assessed the availability and advertising of SSBs in primary schools in Gauteng province two years later and considered whether there were any differences in outcomes based on the socioeconomic status (SES) of the school. In addition, we explored perceptions of school staff around the availability of SSBs in schools and processes related to their implementation of the voluntary pledge.

## Methods

### Study design

A sequential explanatory mixed methods study [27] was undertaken over 14 months between January 2019 and March 2020 in public primary schools in Gauteng province, South Africa. This included quantitative survey of a representative sample of public primary schools’ food environments, followed by a qualitative study among school principals, chairs of school governing bodies (parent-teacher association), and tuck shop managers in a sub-sample of selected schools.

### Study setting and population

The study was conducted in public sector, primary schools. Gauteng is not only highly urbanized but it is considered to be the economic hub of the country and is ranked second highest in terms of the size of school-aged population of the nine provinces [28]. South African public schools are classified into five quintiles of a multidimensional deprivation index with quintile one schools being located in the most deprived areas while quintile five schools are located in the least deprived areas [29]. Schools in quintiles one to three are entirely funded by the state and do not charge fees, while those in quintiles four and five receive less funding from the state and raise further funding by charging fees for attendance.

Operating public primary schools located in Gauteng province registered in the Department of Basic Education Schools’ master-list [30] were eligible for inclusion in the study. Private schools that are not run by the Department of Basic Education and schools that combined primary and secondary phases were excluded as well as schools with missing information on phase, sector, or location in the master-list database. A total of 1 362 schools satisfied the inclusion criteria. Schools were stratified by their fee (quintiles four and five) or no-fee status (quintiles one to three). The stratified sampling design ensured that both fee and no-fee schools were adequately represented within the study sample and allowed us to assess SSB availability and advertising both overall and to examine heterogeneity in the socioeconomic status of schools. Within each stratum, a simple random sample of 65 schools was drawn for a total of 130. Sample size calculations were based on 80% power and 95% confidence and aimed at differences in the satisfaction of the pledge’s commitments in the two samples of the schools. Power calculations were done using the STATA power command.

For the qualitative study, a purposive sample of 26 school principals, heads of school governing bodies and tuck shop managers were selected for in-depth interviews to explore perceptions of the availability of SSBs and other beverages in the schools and processes related to the implementation of the voluntary pledge. The sample ensured maximum variation or diversity and participants were selected based on the presence of school tuck shops or not, whether SSBs were sold or not, geographical diversity and included both fee paying and no-fee schools.

### Data collection

Data on basic school characteristics including geography, district, number of educators and learners were obtained from the school master-list. For measurement of beverage availability and advertising, a team of trained research assistants collected quantitative data based on a guided inspection of the school premises. An observational audit tool was developed using REDCap (Research Electronic Data Capture) [31]. Specifically, the tool included: (1) availability and type of food outlets (tuck shop/informal vendors/vending machines), (2) food and beverage availability in food outlets, (3) advertising and branding (type and size).

Principals of schools were sent an email containing information on the study and explaining the purpose behind administering the food and beverage environment audit tool. After this initial contact, fieldworkers scheduled a convenient day and time for data collection. Principals were requested to give their written informed consent for the observations to be completed on the day of the school visit. They were asked to assign a school representative, usually school administrator, to accompany fieldworkers and walk them around the school premises. Visits to schools were scheduled during school hours when tuck shops were open.

Once the audit tool was complete, semi-structured interviews were conducted with principals, heads of school governing bodies, and tuck shop managers. An interview schedule (see Appendix A) was used as a guide to explore the topic of CCBSA’s voluntary pledge, current school policies on the food environment, perceptions of what food and beverages should or should not be sold in the school premises, any changes in policies or practices relating to the food environment over the past 5 years, the reasons for the changes, and any future plans to further regulate the food environment. Interviews were conducted by two trained research assistants. Interviews were conducted on site at the participating schools and took approximately 30 minutes each. Interviewees were provided with an information sheet that outlined the purpose of the study and the procedures to be undertaken. In-depth interviews were conducted in English, audio recorded, and transcribed. Transcripts were then checked for accuracy by the lead author (AE).

### Measures in the audit tool

School food outlets were assessed for the availability of SSBs and ASBs. Measures were created for four categories: 1) carbonated sugar-sweetened CCBSA products (e.g. Fanta); 2) non-carbonated sugar-sweetened CCBSA products (e.g. Powerade); 3) artificially sweetened CCBSA products (e.g. Coke Zero); and 4) non-CCBSA products (e.g. Pepsi). Advertising and branding were defined as marketing techniques. The former consists of any ‘paid public presentation and promotion of ideas, goods, or services by a sponsor that is intended to bring a product to the attention of consumers through a variety of media channels’, the latter is ‘a marketing feature that provides a name or symbol that legally identifies a company, a single product, or a product line to differentiate it from other companies and products’ [32]. Advertising and branding presence was appraised at locations that included the school entrance, and school tuck shop, as well as sports fields, playgrounds, and swimming pools, if present.

### Data analysis

For the audit data, we constructed estimates of the proportion of schools with CCBSA SSBs and ASBs available and with Coca-Cola products’ branding and advertising present. These proportions were constructed for the population of public primary schools as well as for fee and no-fee schools separately. To test for differences in these outcome proportions across schools’ fee status, we conducted Pearson $\chi^{2}$ tests. 95% CIs were calculated using STATA’s svyset command (to account for survey design) and mean and proportion commands. To account for non-response of schools, we adjusted our weights. Within each strata, weights were the inverse of the probability of selection of a particular school. To adjust for non-response, we multiplied this original weight by the inverse of the probability of non-response we observed within each strata [33].

Qualitative data were analysed and organized thematically. Interview transcripts were independently coded by two researchers (AE, NC) into thematic categories based on deductive (from the interview questions) and inductive (emerging from the transcripts) themes. A codebook was created for data analysis and applied to transcripts. To assess the reliability of the codes, after one round of coding the researchers compared how each segment of the text was interpreted and assessed the level of concordance. Researchers then discussed any disagreement with code application until consensus was reached. Thereafter, transcripts were coded using Nvivo version 12 [34]. Reporting of the findings adheres to COREQ guidelines [35].

### Ethical approval

This study was approved by the University of the Witwatersrand Human Research Ethics Committee (Medical) (#180330) and the Gauteng Department of Education. Prior to data collection, approval and informed consent was obtained from school principals. All interviewees provided written informed consent.

## Results

### Description of participating schools

One-hundred and five schools consented to participation. The response rate was 81% overall. Twenty-five schools refused to participate, and their reasons ranged from the school not wanting to participate in research to the school is going through remodelling. Within Gauteng province, 87% of participating schools were located in urban areas and 40% were from the City of Johannesburg Metropolitan district region (Table 1). The student–teacher ratio was 24.7 in fee schools compared to 29.9 in no-fee schools. Of all schools, 63% (CI: 54–71%) had a tuck shop on their premises, with all fee schools and only 46% (CI: 34–58%) of no-fee schools having a tuck shop. A vending machine was reported in only one school, and none of the schools had a canteen or cafeteria. Approximately half of schools (51%, CI: 41–60%) had a food and beverage outlet within fifty metres of the school premises, with a higher percentage reported around no-fee schools (57%, CI: 45–69%) than compared with fee paying schools (31%, CI: 24–51%).

**Table 1.** Weighted sample characteristics

	All (N = 105)	Fee Schools (N = 44)	No-Fee Schools (N = 61)
Geography (Percentage)			
Rural	13	5	16
	(7–21)	(1–16)	(9–28)
Urban	87	95	84
	(79–93)	(84–99)	(72–91)
District (Percentage)			
City of Johannesburg Metro	40	36	41
	(31–49)	(24–51)	(29–54)
City of Tshwane Metro	22	16	25
	(15–31)	(8–30)	(15–37)
Ekurhuleni Metro	20	23	18
	(13–28)	(13–37)	(10–30)
Sedibeng	11	16	0.08
	(6–18)	(8–30)	(3–18)
West Rand	8	9	8
	(4–16)	(4–22)	(3–18)
Numbers of educators and learners (Mean)			
Number of educators, 2017	31.25	38.74	27.85
	(28.43–34.06)	(32.98–44.49)	(24.64–31.06)
Number of learners, 2017	872.53	957.91	833.51
	(789.89–955.17)	(855.98–1059.84)	(721.20–945.82)
Tuck shop on school premises (Percentage)	63	100	46
	(54–71)	(. – .)	(34–58)
Food and beverage outlets within 50 m (Percentage)	51	36	57
	(41–60)	(24–51)	(45–69)

Notes: 95% confidence intervals in parentheses.

### Quantitative results from audit tool data

#### Availability of CCBSA beverages

Carbonated SSBs were available in 54% (95% CI: 45–63%) of primary schools’ tuck shops, with non-carbonated SSBs were available in 26% (95% CI: 19–35%) (Table 2). Of the non-SSBs, 34% (95% CI: 25–44%) of schools sold carbonated ASBs and 37% (95% CI: 29–46) sold non-flavoured water (Table 3). Across each beverage type, availability was statistically significantly different across fee status of the schools, with CCBSA products generally being more available in fee schools (higher SES). For example, carbonated SSBs were available in 86% (95% CI: 73–94%) of fee schools’ tuck shops versus 21% (95% CI: 10–41%) of no-fee (lower SES) schools’ tuck shops. None of the school tuck shops sold only artificially sweetened CCBSA products. In other words, if a tuck shop did not sell CCBSA SSBs, they also did not stock ASB as an alternative.

**Table 2.** Availability of CCBSA beverages in school tuck shops

	All (N = 72)	Fee Schools (N = 44)	No-Fee Schools (N = 28)	Fee vs No-Fee
Carbonated SSBs (Percentage)				
No	46	14	79	P < 0.01
	(37–55)	(6–27)	(59–90)	
Yes	54	86	21	
	(45–63)	(73–94)	(10–41)	
Carbonated ASBs (Percentage)				
No	66	43	89	P < 0.01
	(56–75)	(30–58)	(70–97)	
Yes	34	57	11	
	(25–44)	(42–70)	(3–30)	
Non-carbonated SSBs (Percentage)				
No	74	52	96	P < 0.01
	(65–81)	(38–66)	(77–100)	
Yes	26	48	4	
	(19–35)	(34–62)	(0–23)	
Any bottled water (Percentage)				
No	63	34	93	P < 0.01
	(54–71)	(22–49)	(74–98)	
Yes	0.37	66	7	
	(29–46)	(51–78)	(2–26)	

Notes: 95% confidence intervals in parentheses. Sample restricted to schools with tuck shops on premises. Pearson $\chi^{2}$ p-value for each outcome in Fee vs No-Fee column.

**Table 3.** Availability of CCBSA ASB products in school tuck shops

	All (N = 72)
Neither carbonated SSBs nor ASBs available (Percentage)	46
	(37–55)
Carbonated SSB available, but not ASBs	20
	(13–30)
Carbonated ASBs available, but not SSBs	0
	(-)
Both carbonated SSBs and ASBs available	34
	(25–44)

Notes: 95% confidence intervals in parentheses. Sample restricted to schools with tuck shops on premises (N = 72).

Table 3 shows that over half of the school tuck shops with sugar-sweetened CCBSA products also sold artificially sweetened CCBSA products.

#### Presence of Coca-Cola branding and advertising

Table 4 shows the presence of Coca-Cola branding or advertising on school premises. Nearly one-third (31%; 95% CI: 73–94%) of schools had Coca-Cola branding or advertising on school premises, with 23% (95% CI: 18–28%) of schools having tuck shop advertising and 10% (95% CI: 6–18%) with school entrance advertising. Fee paying (higher SES) schools were more likely to have tuck shop branding and advertising present than compared with no-fee paying (lower SES) schools but were equally likely to have entrance advertising.

**Table 4.** Advertising of Coca-Cola products on school premises (n = 105)

	All (N = 105)	Fee Schools (N = 44)	No-Fee Schools (N = 61)	Fee vs No-Fee
Any Coca-Cola advertising (Percentage)				
No	69	30	87	P < 0.01
	(61–75)	(18–44)	(76–93)	
Yes	31	70	13	
	(25–39)	(56–82)	(7–24)	
Any tuck shop Coca-Cola advertising (Percentage)				
No	77	36	97	P < 0.01
	(72–82)	(24–51)	(88–99)	
Yes	23	64	3	
	(18–28)	(49–76)	(1–12)	
Any school entrance Coca-Cola advertising (Percentage)				
No	90	89	90	P = 0.80
	(82–94)	(76–95)	(80–95)	
Yes	10	11	10	
	(6–18)	(5–24)	(5–20)	

Notes: 95% confidence intervals in parentheses. Presence of tuck shop advertising coded as zero if school did not have tuck shop. Pearson $\chi^{2}$ p-value for each outcome in Fee vs No-Fee column.

### Qualitative findings from in-depth interviews

#### Awareness of the pledge

We asked principals, tuck shop managers and school governing body representatives if they were aware of CCBSA’s pledge. Ten of the 26 participants recalled either receiving a notice from CCBSA or remembered a CCBSA sales representative visiting the school to inform them about the pledge. Participants who remembered receiving the notice understood that CCBSA would no longer be selling SSBs.

They [CCBSA representatives] came along we had to sign papers […] just to say that we can’t get sugared cold drinks anymore only diet cold drinks and they said they going to deliver the sugar free products and then latter they came and gave us papers. (School 23, Q5, fee paying)

Despite a general sense of some sort of restrictions that were included in the notice that they received, participants could not recall the specifics of the pledge and expressed confusion. For instance, one of the principals misinterpreted the content of the notice as a restriction on the supply of fizzy drinks rather than the sugar content and reported the availability of other sugar-sweetened CCBSA products such as sweetened iced teas and energy drinks at schools.

What they said due to the sugar tax, that they are going to stop supplying all primary schools with their product, they only had the diet cold drinks and of course things like Powerade. (School 7, Q5, fee paying)

#### Reasons for limited availability of SSBs

In schools without SSBs, respondents attributed this to the low-socioeconomic status of the area and the lack of school tuck shops. Participants explained that students could not afford to buy food and beverages at school and as a result, a tuck shop could not be supported. This finding was supported by the quantitative data that showed that 82% of no-fee paying (lower SES) schools did not have a tuck shop and only 19% of no-fee paying schools had sugar-sweetened CCBSA products on sale. Interviewees indicated that their students have little tuck shop money. They explained that students either did not have money to buy SSBs at all, or when they did have money, they could not afford CCBSA products due to the higher prices when compared to other brands.

No, we can’t [sell SSBs] because you know primaries [primary school students] they just want thing for R2/R1 [US$ 0,11/0,054] (School 18, Q5, fee paying)

We never kept Coca-Colas specifically for the kids because we felt it was too high priced in terms of the market, in terms of the area and from the level of finances they come from. So we kept more budget like Pepsi because is R8.00 [US$ 0,46] (School 10, Q5, fee paying)

In schools where SSBs were available to some extent, participants described school-level restrictions around selling sugar-sweetened products only at specific times of the day, for example, prohibiting the consumption of any sugar during the morning school breaks, including both drinks and sweets. Participants also spoke of restrictions based on age with some schools restricting sales of SSBs to teachers, parents and visitors, while others prohibiting SSBs for students in grades 1–3 (children aged 6–9 years).

I informed the juniors, Grade 1-3 [students], I told them that they are not allowed to actually go and purchase soft drinks as well as sweets. (School 9, Q5, fee paying)

Kingsley [carbonated SSB] for the kids is not right so they took it out [from tuck shops]. Now, we are selling the proper juice [for the students] and the cold drinks the Coca-Cola, Fanta for the teachers. (School 15, Q5, fee paying)

In some instances, there were restrictions on the sale of SSBs, which participants attributed to links between their intake and students’ behaviour. Too much sugar was linked with low concentration levels in the classroom, an issue that teachers were particularly concerned about.

We have also indicated that in the mornings when children are coming to school, no sweet stuff [candy, SSBs] should be sold to the learners because we found that when they are in the classrooms, they don’t concentrate. (School 6, Q5, fee paying).

The harmful health consequences of drinking SSBs including obesity were also cited by participants as a reason to restrict SSB sales and consumption levels.

There are few things which we [school directorate] negotiate that she [tuck shop owner] doesn’t sell like, coca cola because of the fact that some parents raised issues of obesity. We are trying to avoid that. (School 13, Q2, no-fee)

#### Reasons for availability of SSBs

Respondents’ most common reasons for SSB availability in schools included demand and profit generation both from the tuck shop perspective and fundraising purposes for the school.

If you don’t have Coca-Cola in your fridge your customers go [lose customers]. (School 8, Q5, fee paying)

Interviewees explained that tuck shop operators must trade-off income generation against the healthfulness of the products they sell.

It is a balancing act because you want to focus on the healthy foods, but you also realise that you get a good form of income by the soft drinks” (School 9, Q5, fee paying).

From the tuck shop point of view as I explained this is a profit making business and she [the tuck shop owner] is going to sell what the children want in order for her to survive because she pays the school a rent so she gone sell the wrong things [SSBs]. (School 06, Q5, fee paying)

When participants discussed ASBs like Coca-Cola Zero, they claimed that these were less popular among students.

I have the zeros [zero sugar drinks] but for the teachers, the kids don’t buy zeros … they want the real thing [regular SSB] (School 25, Q5, fee paying)

We only have cold drinks because we tried fruit juices in the past and expiring date has come and things get wasted so we don’t do fruit juice we only [sell] cold drinks and things like PowerAde and water when [it] is our sports [event] (School 07, Q5, fee paying)

Cold drinks, kids love it. We did try fruit juices and things like that. It did not go well, anything healthy the kids were like ‘no we don’t want’. You can give guidelines and give suggestions, but you can’t force [the consumption of healthy foods and beverages]. (School 01, Q5, fee paying)

Interviewees indicated that they preferred to have a variety of products on sale, including SSBs.

I am not going to sell [SSBs] to students because I am obeying the rules, it is hard that I have [SSBs on sale] in front of them. I have been sticking to it you can ask the kids they were miserable with it but they are used to it. Dealing with the parents and other adults is a bit difficult because they don’t understand so I do keep [SSBs] like there and if I feel I can sell to you then I will sell to you. (School 14, Q5, fee paying)

Some respondents reported restrictions on SSB sales as ineffective because of the easy access to unhealthy products outside of school premises. The potential benefits achieved by the school through restriction would be hindered by unhealthy dietary habits at students’ households, as well as purchases by students from informal vendors and corner shops, in close proximity to the schools.

When ABI [former distributer of CCBSA] stopped delivering to us he said, ‘they can go around the corner to the first Spar or cafe and buy cold drink’ so it didn’t make sense what ABI and the department was trying to do because every home in South Africa is drinking cold drink so to deny the child from drinking cold drink here [in school] but they are going home to have it, it didn’t make sense. (School 7, Q5, fee paying)

#### Changes to SSB supply, sales and promotion in schools

When asked about any SSB-related changes that the school experienced over the past years, participants discussed changes to the suppliers, to the sugar content of the drinks that were being sold, as well as changes to branding and advertising on the school premises. Many participants indicated that the primary process of change was driven by the suppliers who provided some information about the new policies around the supply of SSBs. Most respondents referred to CCBSA as their main supplier, while some cited ABI (Amalgamated Beverage Industries), the former distributer of CCBSA products. Three schools reported receiving a notice from the Department of Basic Education rather than from CCBSA. Participants indicated that following the notice, their contact with sales representatives diminished significantly.

They are no longer coming like they used to come like I indicated that they would come maybe twice per week. But now I can spend the whole month without to seeing even one person from Coca-Cola. (School 26, Q3, no-fee)

Participants shared their experience of how the interruption in the supply of SSBs and the reduced interaction with CCBSA representatives prompted them to look for alternative suppliers.

I think it’s about two years ago they [CCBSA] had their sugar drive and then they told us we can only buy the Zeros, the waters and the Energades and then most of our tuck shop owners said ‘you know what we cannot, I buy like 1 or 2 or 3 cases [of ASBs] a month maybe for each shop the rest is still the other stuff, the normal sugar stuff [regular SSBs]’. It was such a problem for us because now you cannot get your whole variety from them so we had to source different places. (School 8, Q5, fee paying)

Regarding advertising, some schools reported that CCBSA has done what was promised in the pledge, to remove all branding and advertising from schools. However, this was the exception rather than the norm. Respondents reported that branding, in particular, branded fridges remained unchanged. Certain interviewees welcomed the remaining branded fridges as a valuable asset for the schools, while some respondents perceived branding as a challenge.

I had the Coca-Cola account that is why I felt like maybe targeted because they had me on record as a primary school selling Coca-Cola and ordering Coca-Cola every week, because now, I haven’t seen the rep in about two years, he used to come to me all the time, regular on the Tuesday he here for his order, now I haven’t seen him ever since. They haven’t changed the signages which I think is so unfair because we can’t sell it but is still in there. (School 14, Q5, fee paying)

## Discussion

In this study, we examined a recent example of a voluntary initiative where a large beverage entity, CCBSA, pledged to no longer sell and advertise their products on primary school premises. We found that while the wording of the CCBSA pledge indicated their intention to replace SSBs with ASBs and remove branding and advertising from primary schools, their SSB brands remained available in a majority of public primary schools which had tuck shops. However, there was some heterogeneity in this availability by socioeconomic status. Firstly, higher SES public primary schools were significantly more likely to have tuck shops where such products might be available and advertised. And secondly, focusing only on schools, which had tuck shops, lower SES schools were much less likely to have SSBs available. Interviews with school decision-makers attributed this to the lower incomes of students at no-fee schools which was perceived to constrain demand. Adding further complexity, we found that no-fee schools were more likely to have food and beverage outlets near their perimeter.

There were several reasons why the pledge may not have resulted in the removal of CCBSA brands and advertising from school environments. Firstly, not all school decision-makers were aware of the pledge suggesting that they either did not recall the letter or had not seen it. Whether or not schools received the letter outlining the pledge, the low awareness limits the potential impact of the pledge to address children’s consumption of SSBs. Secondly, CCBSA’s commitment relied on their sales team removing/replacing branding and advertising from primary schools only after specifically requested to do so by the school. The pledge thus assumed that schools (i) knew about the pledge, and (ii) were motivated enough to act on the pledge and request CCBSA to change existing branding and advertising and to remove fridges. For a majority of schools, the branded fridges were the only ones available and so the incentive to remove them was low. Moreover, school personnels had limited knowledge of the harms of SSBs. They also faced competing priorities such as the need for tuck shop revenue for general school maintenance and staff salaries. Finally, our interviews revealed that CCBSA is not the sole distributer to school tuck shops. Some schools stocked their tuck shops through general commercial wholesalers.

The finding of continued availability and advertising of CCBSA brands despite the voluntary pledge is not unique. There is a history of voluntary pledges being implemented in many different industries globally with limited success. A systematic review of over a hundred studies of the alcohol industry’s voluntary marketing pledges found that codes are routinely violated and expose vulnerable populations including children to harmful advertising of alcohol [36]. Self-regulatory systems have been proven ineffective at preventing code violations in alcohol advertising in Brazil, where adolescents and experts found popular beer television advertisements to violate the code of targeting adolescents and children [37], and in Ghanaian television where the spirits and beers were marketed with animated bottles and cartoon characters play football, thereby attracting children’s attention [38].

While our study reported on the implementation of the pledge in schools, the obesogenic environment extends outside the school premises [13], and into households. If the South African government wants to address children’s consumption of SSBs and other ultra-processed foods, there is a need to tackle broader environmental and socioeconomic factors associated with in- and out-of-school beverage intake and choices. Our findings of difference in the prevalence of tuck shops and SSB availability and advertising by schools’ SES suggest that solely intervening in school environments could produce perverse equity outcomes, with the benefits of such policies accruing to students from schools located in neighbourhoods with higher SES.

These results emphasise the importance of raising awareness about the link between sugar and obesity-related diseases. We found evidence for confusion among school staff whose priority issue was sugar-induced hyperactivity and paid less attention to the link between sugar and obesity. Nutrition knowledge of school staff is critical to promoting healthy food habits [39] and effective communication strategies are necessary to improve understanding of the link between sugar and SSBs.

Comprehensive, compulsory regulatory action is likely needed to reduce the availability of SSBs to children to ensure that healthy choices are made [40,41]. One key public policy especially for children is to regulate the sale and marketing of SSBs in primary schools. Policy options include banning the sale and advertisement of SSBs on school property [42,43]. Explicit steps should also be taken to monitor compliance and violations should be treated seriously and the companies should be charged accordingly [44]. These could include establishing linkages between the National Department of Health and Education and the National Consumer Commission to encourage whistleblowing on companies that transgress regulations on inappropriate marketing of food to children.

## Limitations

Firstly, the study was cross-sectional in nature and we do not have a baseline that preceded the voluntary pledge by CCBSA. Secondly, it is also unclear if schools had made any changes to the food environment between the announcement of our visit and administering the audit tool. To limit any potential source for social desirability bias, the audit tool was completed by our research staff rather than being self-administered. Moreover, it is possible that recall bias may have affected how some participants responded to questions about the pledge. Thirdly, we did not examine the extent to which the school environmental factors influenced dietary intake as this study focussed on availability as an important first step in ensuring a healthier nutritional environment. Finally, it is possible that differential response rate could have introduced some sampling bias as a greater proportion of fee paying schools refused to participate. Since fee schools were more likely to have tuck shops and to sell SSBs however, it is likely that their inclusion would strengthen the findings of the study which were that there was continued availability of SSBs.

## Conclusion

Profit-driven corporate actors are important contributors to unhealthy food environments. A common activity that corporations undertake is pledging to voluntarily restricting marketing and sales of their products to children. In the context of South Africa’s nutrition transition and rising childhood obesity, we found that despite a voluntary pledge by CCBSA, SSBs continued to be sold and advertised in a significant proportion of schools two years after its announcement. These findings and those of the broader literature on voluntary pledges suggest that mandatory regulation may be required to restrict the sale of SSBs in primary schools. However, a one size fits all approach may not be sufficient, in particular, low SES schools may have very different food environments and may face a different set of challenges in the realization of healthy food environments. Policies need to be introduced that restrict how corporate actors shape the food environment, especially those that affect children.

## Data Availability

The dataset analyzed during the study is available from the corresponding author on reasonable request.

## Paper context

Among the drivers of South Africa’s obesity epidemic are sugar-sweetened beverages (SSBs). To avoid government regulations, corporations often pledge to restricting marketing and sales of their products to school-age children. The limited success in the history of voluntary pledges casts doubt on their impact. This paper examines the availability and advertising of SSBs in primary schools, following a pledge by a beverage entity in SA to restrict marketing and sales of SSBs on school premises.

## Appendix A. Semi-structured interview guide

**A: Policies and Guidelines related to the Food Environment**

1. Tell me about the school policies and guidelines that guide what food and drinks are available inside the school premises
  - How did the policies come about? (who was involved and what was the process)
  - What were the reasons behind the development of any policies or guidelines?
  - What do the policies cover? (tuck shops, feeding programme)
2. How well are any policies and guidelines being implemented
  - What are the barriers to implementation?
  - What are the facilitators of implementation?

**B: Food and Beverage Environment**

3. What kind of drinks are available to children in the school?
4. What kind of food and drinks are available to learners directly outside the school?
  - How do children access the food and drink?
5. What are your thoughts about the food and drinks that should be available in the school?
  - What are your reasons for your views
6. What makes it difficult in the school to make changes to improve the nutrition of students?
7. What nutrition-related support do students in the school currently have?
  - Who are the main supporters involved?
8. What nutrition-specific activities happen at the school (if any at all)
  - What other nutrition-specific activities would you like to see happening?

**C: Coca-Cola’s voluntary pledge**

9. What contact have you had with Coca-Cola or any of their representatives, if at all?
  - What do you know about the voluntary pledge that Coca-Cola has made (if anything at all)
  - What contact have you had with any other soft drink representatives (other than Coca-Cola)?

**D: Other**

10. To what extent do you think that the wealth quintile classification of the school represents the learners
11. What are the school’s plans for the future to address issues of nutrition?
12. Please share any other information that I have not asked about specifically that you think is important?
